# The correlation of EZH2 expression with the progression and prognosis of hepatocellular carcinoma

**DOI:** 10.1186/s12865-022-00502-7

**Published:** 2022-06-04

**Authors:** Shi-yi Wu, Zhao-yu Xie, Lu-yu Yan, Xiao-fang Liu, Yue Zhang, Da-an Wang, Jie Dong, Hong-tao Sun

**Affiliations:** 1 Department of Cardiology, Inner Mongolia University for Nationalities Second Clinical Medical College, Lincheng Road, Yakeshi, 022150 China; 2 Ophthalmology Department, The Second Xiangya Hospital, Central South University, No. 139, Renmin Middle Road, Changsha, 410000 China; 3 Department of Endocrinology, Inner Mongolia University for Nationalities Second Clinical Medical College, Lincheng Road, Yakeshi, 022150 China; 4 Department of Gastroenterology, Inner Mongolia University for Nationalities Second Clinical Medical College, Lincheng Road, Yakeshi, 022150 China; 5 Department of Cardiology, Affiliated Hospital of Inner Mongolia University for Nationalities, Tongliao, 028000 China

**Keywords:** Immunotherapy, Immune infiltration, Liver hepatocellular carcinoma, T cells exhaustion, Prognosis, EZH2

## Abstract

**Background:**

Enhancer of Zeste homologue 2 (EZH2) is a polycomb group gene and an epigenetic regulator that inhibits transcription, a modification associated with gene silencing. EZH2 plays an essential role in humoral and cell-mediated adaptive immunity. The purpose of the current study is to investigate the prognostic potential of EZH2 and to comprehensively analyse the correlation between EZH2 and immune infiltration in multiple cancer cases, especially liver hepatocellular carcinoma.

**Methods:**

EZH2 expression across cancers was explored through Oncomine, HPA, and GEPIA2. Additionally, the prognostic value of EZH2 analysis across cancers was based on the GEPIA2, TCGA portal, Kaplan–Meier Plotter, and LOGpc databases. Based on GO and KEGG analyses, GSEA helped demonstrate the biological processes through which EZH2 might lead to HCC development. GEPIA and TIMER were adopted to detect the possible relationship of EZH2 expression with tumour-infiltrating immune cells (TIICs).

**Results:**

EZH2 overexpression levels were associated with poor prognosis of cancer, especially hepatocellular carcinoma. A high EZH2 expression level is related to a poor prognosis of HCC, especially in disease histology and stage III. The EZH2 expression level was positively correlated with critical gene markers of TAMs, M2 macrophages, M1 macrophages, and monocytes. Further analysis revealed that EZH2 genes were mainly related to DNA recombination, mitotic cell cycle phase transition, and chromosome segregation.

**Conclusion:**

EZH2 plays an essential role in the immune microenvironment and is a potential prognostic marker and immunotherapy target for hepatocellular carcinoma.

**Supplementary Information:**

The online version contains supplementary material available at 10.1186/s12865-022-00502-7.

## Introduction

Immunotherapy for cancer is becoming a critical approach that manages cancer cells via the immune system [1–3]. Several studies in some preclinical models and advanced tumour patients have proven that epigenetic modulators have immunomodulatory properties, providing a theoretical basis for combining epigenetics with immunotherapy [4–6]. Liver hepatocellular carcinoma (LIHC) is the most common cancer globally, and it is the third primary cause of tumour-related deaths [7, 8]. Over the past decade, efforts have been made to develop novel drugs and therapeutic strategies for HCC [9, 10]. However, the efficacy of anti-LIHC therapy is compromised due to unclear carcinogenesis and progression mechanisms at the molecular level [11]. Serum alpha-fetoprotein (AFP) detection, B ultrasound, and CT scans can be used to diagnose liver cancer. However, the misdiagnosis rate is high [12, 13]. Currently, biomarkers for liver cancer are rapidly advancing, but the 5-year survival remains low [14, 15]. Therefore, more sensitive biomarkers and novel therapeutic strategies must be explored for HCC treatment.

EZH2 is a polycomb group gene (PcG) and an epigenetic regulator that inhibits transcription. Polycomb repression complex 2 (PRC2) in the PcG protein core complex can regulate chromatin structure-mediated gene silencing [16]. EZH2 acts as an enzyme catalytic subunit of PRC2, mediating trimethylation of Lys27 in histone 3 (H3K27me3) and gene silencing [17]. H3K27me3 inhibits gene expression [18]. In addition to H3K27me3, PRC2 also methylates nonhistone substrates [19]. EZH2 activates downstream genes by methylating nonhistone targets in a PRC2-independent manner [20–22]. As a result, EZH2 crucially affects cell lineage determination and signalling pathways and is a master regulator of cell cycle progression, autophagy, apoptosis, DNA damage repair, and cellular senescence inhibition [23–26]. Studies have revealed that EZH2, a modifier associated with epigenetic regulation and immune function, can promote local and systemic anticancer immune responses by modulating TH-1 chemokine expression, affecting marrow-derived suppressor cells (MDSCs) or CD8 + T-cell infiltration [27–31]. However, the mechanisms of human tumours must be explained. These pieces of evidence indicate that EZH2 may be a modifier involved in epigenetic regulation and immune function.

The work presented in the current study provides an analysis of the role of EZH2 levels in LIHC and explores the function of EZH2 in tumour immunity, which was derived from publicly accessible databases. Our study confirmed that EZH2 upregulation could predict poor overall survival (OS) in HCC patients. This illustrated the possible association and regulatory mechanism of EZH2-associated genomic alterations and functional networks within LIHC and helped identify novel diagnostic and therapeutic LIHC targets.

## Methods

### Oncomine analysis

EZH2 gene expression levels in tumour and normal tissues was obtained from the Oncomine database (http://www.oncomine.org), a web-based data mining platform for collecting, analysing, and offering tumour microarray information [32].

### Comprehensive correlation analysis in tumour-infiltrating immune cells

Tumour Immune Estimation Resource (TIMER), a free database containing 32 TCGA-derived cancers involving 10,897 samples, can assess inner immune infiltrate levels (http://cistrome.org/TIMER/). We evaluated the association of EZH2 levels with six immune cell types within LIHC using the TIMER database [33–36]. Similarly, we investigated the association of EZH2 expression with tumour purity.

### Analysis based on the OnCoLnc Database

The OncoLnc database contains 21 pieces of TCGA cancer survival information (http://www.oncolnc.org/). In addition, the current study analysed EZH2’s prognostic value in 21 cancers. In accordance with the obtained results, the EZH2 expression level was remarkably related to survival in eight cancers.

### Gene expression profiling interactive analysis

GEPIA2 (The Gene Expression Profiling Interactive Analysis 2) database is a comprehensive analytical tool that analyses customizable functions, such as the interaction function and genes’ prognostic significance in cancer and noncarcinoma samples [37] (http://gepia2.cancer-pku.cn/). The current work used GEPIA to detect EZH2 mRNA levels in LIHC and its prognostic value and analysed gene expression correlations.

### Kaplan–Meier (K–M) survival curve analysis

K-M Plotter can be adopted for evaluating the connection between gene expression and 21 cancer prognoses (http://kmplot.com/). In the current study, we adopted K-M Plotter to detect the connection between EZH2 levels and HRs, P values (upon log-rank test), OS, and RFS in LIHC patients. More than 50,000 samples from the database were evaluated using gene array and RNA sequencing [38].

### TISIDB database analysis

TISIDB is a public database used for analysing the interactions between the immune system and cancers (http://cis.hku.hk/TISIDB). It combines different cancer immunology data sources [39]. Based on the TISIDB database, we explored the Spearman correlation between EZH2 expression level and tumour-infiltrating cell level and subtype.

### Human protein atlas database analysis

The HPA database includes the gene expression profiles and pathology, which can be adopted to validate prognostic gene immunohistochemistry (IHC) [40] (https://www.proteinatlas.org/). IHC images from HPA can be directly accessed at EZH2 in tumour tissue (https://www.proteinatlas.org/ENSG00000148773-EZH2/pathology/liver+cancer#img) and EZH2 in normal tissue (https://www.proteinatlas.org/ENSG00000148773-EZH2/tissue/liver).

### Coexpression gene prediction and GSEA

LinkedOmics is an open platform that includes multiomics data for 32 cancers derived from TCGA (http://www.linkedomics.org/) [41]. We screened for EZH2-related differentially expressed genes in the TCGA LIHC cohort (n = 371) using LinkFinder's LinkedOmics, and their correlations were analysed using Pearson's correlation. The LinkInterpreter module was employed for network and pathway analyses of DEGs. Kyoto Encyclopedia of Genes and Genomes is a database (https://www.kegg.jp/) that relates genomic information to higher-order functional information for analysing gene function [42–44]. KEGG pathway and GO analyses were performed using GSEA tools.

## Results

### Expression level of EZH2 across cancers

**Fig. 1:**
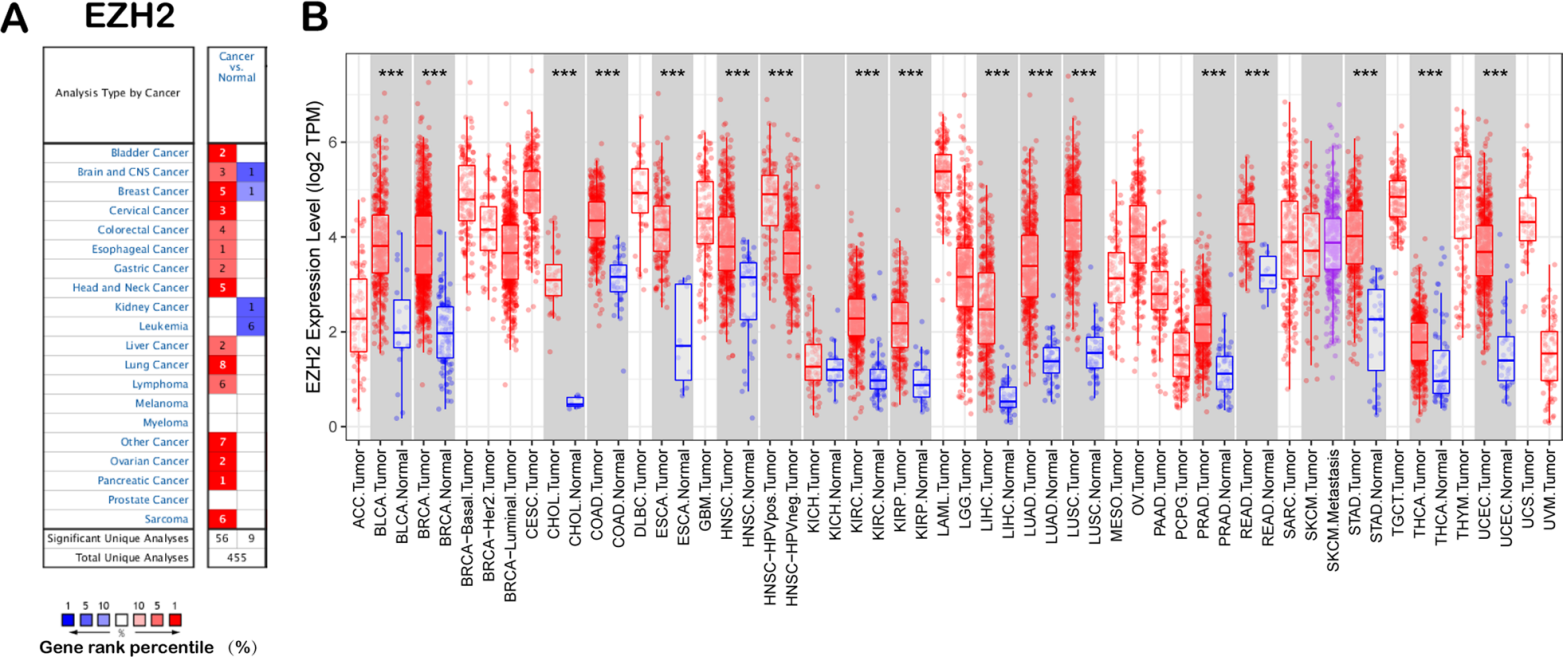
EZH2 levels within pan-cancers. **A** High or low EZH2 expression within pan-cancers relative to healthy samples from the Oncomine database. **B** EZH2 levels within pan-cancers in TCGA database measured through TIMER. *P < 0.01, **P < 0.001, ***P < 0.0001

The current study used Oncomine database to analyse the difference in EZH2 expression between carcinomas and normal samples. Our analysis revealed that EZH2 is highly expressed in breast, bladder, head and neck, sarcoma, pancreatic, cervical, liver, and other cancers compared with in normal tissues (Fig. 1A). Additionally, EZH2 downregulation was detected in prostate cancer, myeloma, melanoma, kidney cancer, and leukaemia cancers. Additional file 2: Table S1 offers more detailed results of EZH2 expression across cancers. TIMER was used to detect RNA sequencing data in TCGA to evaluate EZH2 expression across cancers. Differential EZH2 expression between cancer and healthy samples is displayed in Fig. 1B. EZH2 expression in SKCM was significantly lower than that in healthy samples. However, EZH2 expression was upregulated within the BRCA, BLCA, COAD, CHOL, HNSC, ESCA, KIRP, KIRC, KICH, LIHC, PRAD, LUSC, LUAD, STAD, READ, UCEC, and THCA.

### Prognostic potential value of EZH2 across cancers

**Fig. 2:**
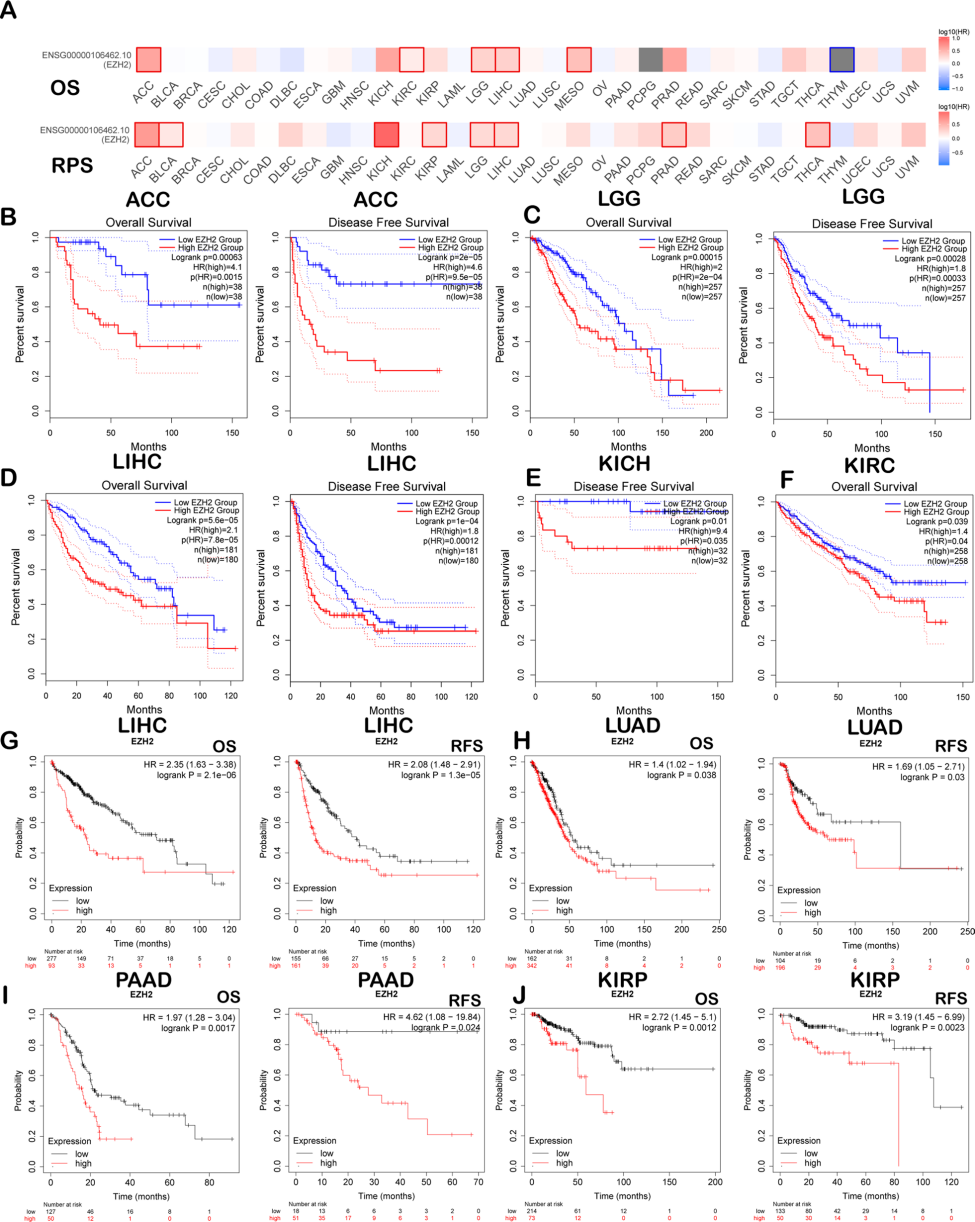
Comparison of EZH2 expression within diverse cancers (A–F) by K–M survival curves based on GEPIA2 database as well as K–M Plot (G–J). **A** Survival heatmap showing EZH2 within 33 cancers obtained from the TCGA database. The rectangular box indicates the significance of prognostic analyses, and the blue and red squares indicate low and high expression, respectively. DFS and OS curves for **B** ACC (n = 38), **C** LGG (n = 257), and **D** LIHC (n = 180); DFS curves for **E** KICH (n = 32); and OS curves for **F** KIRC (n = 258). OS and PFS survival curves in **G** LIHC (n = 370, n = 316), **H** LUAD (n = 504, n = 300), **I** PAAD (n = 177, n = 69), and **J** KIRP (n = 287, n = 183). OS-overall survival, RFS-relapse-free survival, DFS-disease-free survival, and PFS-progression-free survival

The current study analysed EZH2’s effect on cancers prognosis from three databases. Table 1 presents the correlation of EZH2 levels with cancers survival from the OncoLnc database. We assessed the correlation between EZH2 expression level and prognosis by using GEPIA2. Figure 2A presents the influence of EZH2 levels on survival in cancer. Poor prognosis was associated with high EZH2 expression levels in ACC (OS: (P = 6.3e-04, HR = 2); PFS: P = 2.8e-04, HR = 1.8); LGG (OS: P = 1.5e-04, HR = 2); PFS: P = 2.8e-04, HR = 1.8); LIHC (OS: P = 5.6e-5, HR = 2.1); PFS: P = 1.0e-04, HR = 1.8); KICH (OS: P = 0.01, HR = 9.8); and KIRC (OS: P = 0.039, HR = 1.4) (Fig. 2B–F). Then, we assessed EZH2-related survival (OS and RFS) by using Kaplan–Meier Plotter. Similarly, we discovered that EZH2 is a detrimental prognostic factor for LIHC (OS: P = 2.1e-06, HR = 2.35 [1.63–3.38]; PFS: P = 1.3e-05, HR = 2.08 [1.48–2.91]), LUAD (OS: P = 0.038, HR = 1.4 [1.02–1.94]; PFS: P = 0.03, HR = 1.69 [1.05–2.71]), PAAD (OS: P = 1.7e-03, HR = 1.97 [1.28–3.04]; PFS: P = 0.024, HR = 4.62 [1.08–19.84]), KIRP (OS: P = 1.2e-03, HR = 2.72 [1.45–5.1]; and PFS: P = 2.3e-03, HR = 3.19 [1.45–6.99]) (Fig. 2G–J).

**Table 1:** Prognostic role of EZH2 in diverse cancers through OncoLnc

Cancer	Cox	*P*-value	FDR	Rank	Median	Mean
BLCA	0.032	7.00e-01	8.58e-01	13,285	475.71	599.61
BRCA	0.082	3.50e-01	7.03e-01	8182	395.74	504.19
CESC	− 0.327	1.60e-02	2.05e-01	1239	1033.8	1118.51
COAD	− 0.147	1.50e-01	5.58e-01	4290	657.07	697.51
ESCA	0.001	9.90e-01	9.95e-01	16,624	482.73	573.25
GBM	− 0.037	6.90e-01	9.58e-01	12,003	598.43	689.2
HNSC	− 0.158	2.40e-02	2.46e-01	1590	479.81	577.87
KIRC	0.292	2.30e-04	1.62e-03	2346	126.98	149.04
KIRP	0.527	7.90e-04	8.76e-03	1477	123.29	140.76
LAML	− 0.028	8.20e-01	9.43e-01	13,200	1041.46	1043.88
LGG	0.277	6.20e-03	1.62e-02	6414	216.22	286.97
LIHC	0.478	2.80e-06	1.23e-03	36	248.55	306.71
LUAD	0.097	1.80e-01	4.10e-01	7248	320.93	397.7
LUSC	− 0.088	1.90e-01	7.01e-01	4467	629.89	713.28
OV	− 0.003	9.70e-01	9.93e-01	16,448	503.56	601.14
PAAD	0.228	4.10e-02	1.63e-01	4299	207.07	238.52
READ	− 0.188	3.80e-01	9.57e-01	6463	643.34	675.6
SARC	0.133	2.00e-01	4.83e-01	6586	440.14	578.62
SKCM	0.052	4.40e-01	6.36e-01	11,030	447.42	528.58
STAD	− 0.169	5.30e-02	3.49e-01	2548	483.32	521.53
UCEC	0.121	3.00e-01	9.88e-01	4955	460.41	528.01

Significantly different results are displayed in this table (*P* < 0.05)

### Relationship between EZH2 expression and Immune Infiltration

**Fig. 3:**
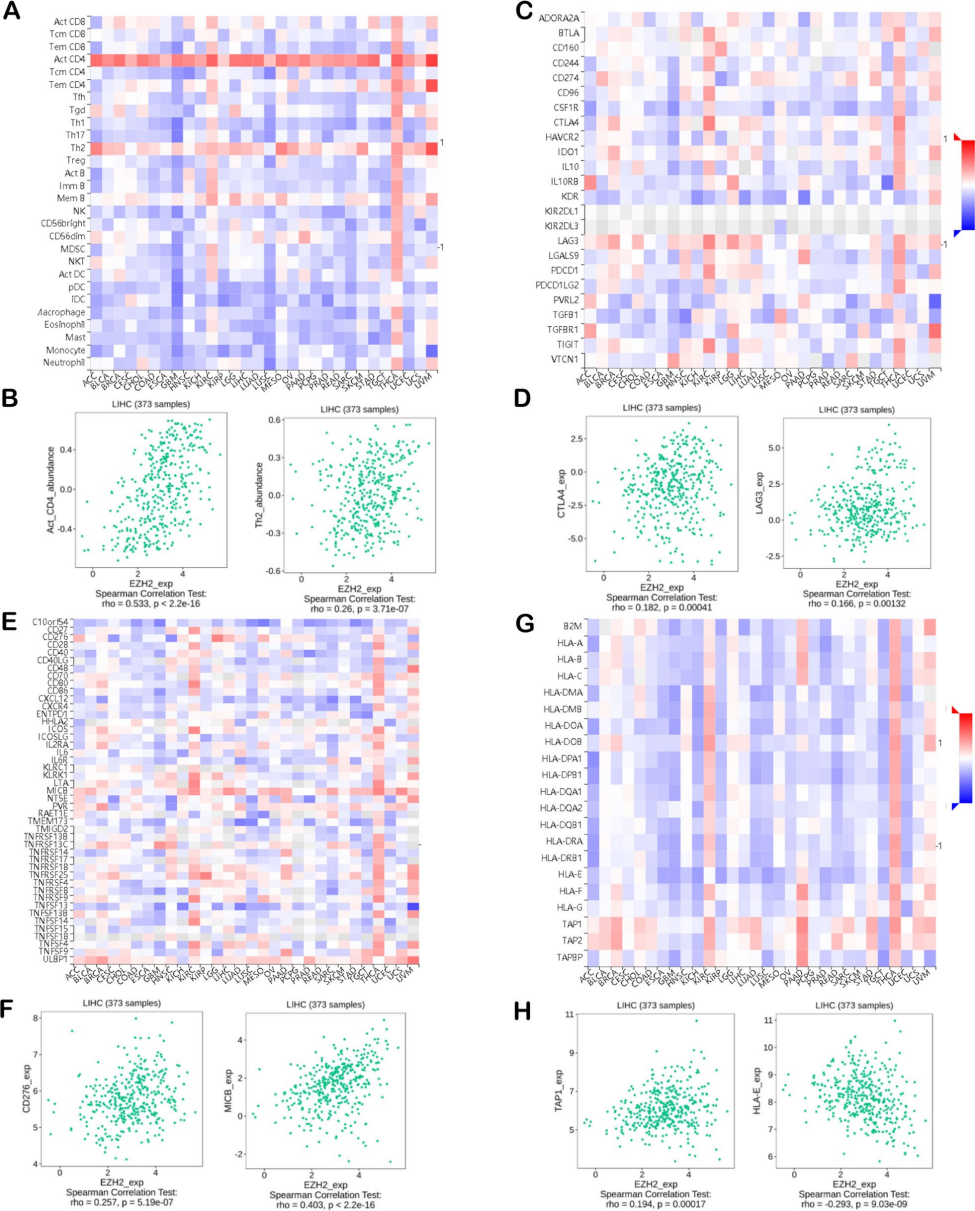
Spearman’s correlation for the EZH2 level and TIIC infiltrating degrees (TISIDB). **A** Connection of TILs levels with EZH2 expression. **B** In Spearman analysis, acT-CD4 and Th2 of TIL has the strongest correlation with the EZH2 level. **C** Relationship between immunosuppressant and the EZH2 level. **D** In Spearman analysis, CTLA4 and LAG3 of immune inhibitors has the strongest correlation with the EZH2 level. **E** Relationship between immunostimulators and the EZH2 level. **F** In Spearman analysis, CD276 and MICB of immunostimulators has the strongest correlation with EZH2 level; **G** Association of MHC molecules with the EZH2 level. **H** In Spearman analysis, TAP1 and HLA-E of MHC molecules has the strongest correlation with the EZH2 level. Negative and positive correlations are represented by blue and red, respectively. Colour intensity is directly proportional to correlation strength. MHC-major histocompatibility complex; TILs-tumour-infiltrating lymphocytes

**Fig. 4:**
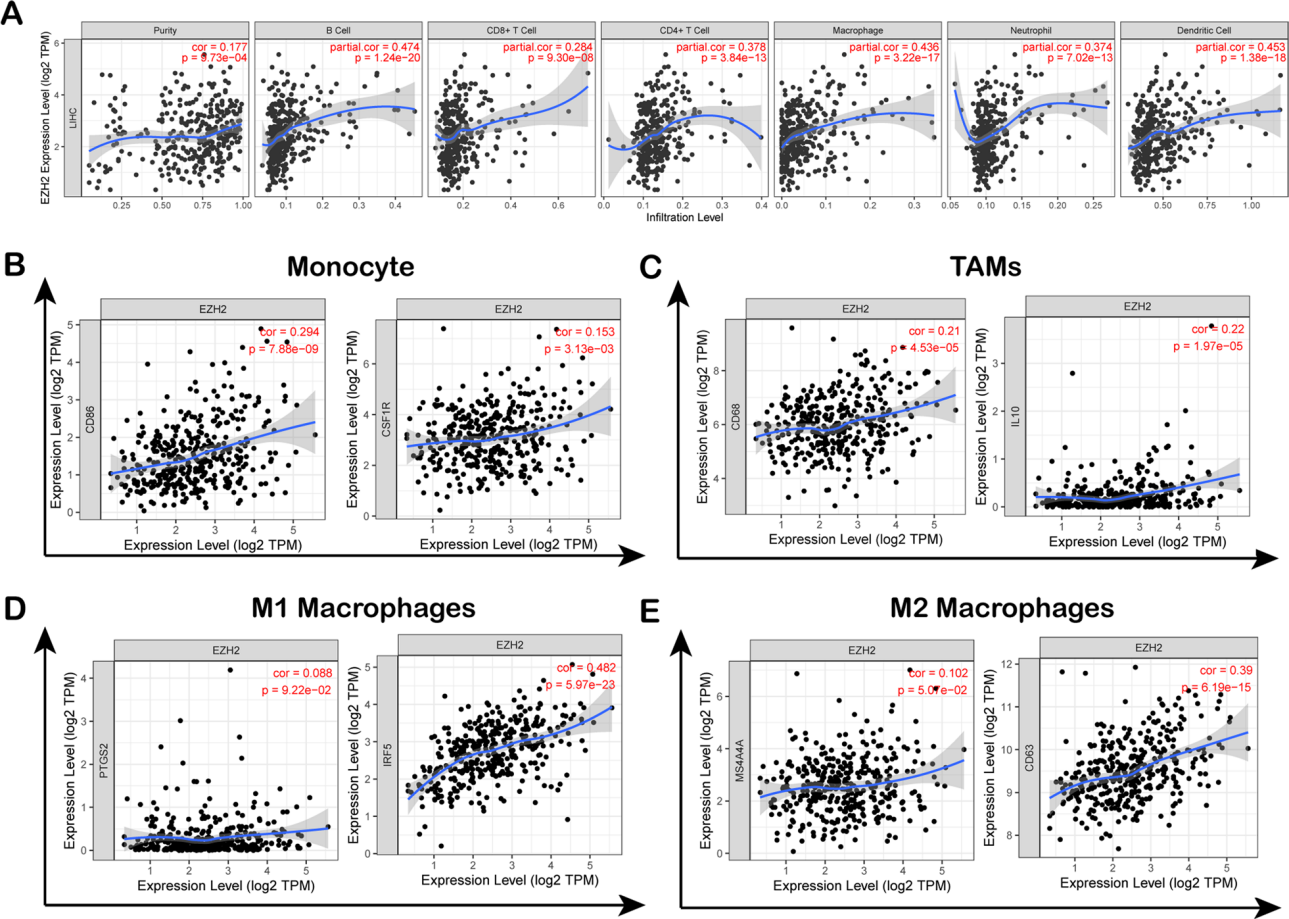
Connection between EZH2 and TIIC infiltrating levels in LIHC. **A** EZH2 expression showed a positive relation with infiltrating degrees of CD8 + T cells, B cells, neutrophils macrophages, and DCs within LIHC based on TIMER database; **B**–**E** Scatter plots showing the connection of EZH2 expression with TIIC markers

Cancer survival and lymph node metastasis (LNM) might be predicted according to tumour-infiltrating immune cell (TIIC) levels [45–47]. The current study analysed the connection between EZH2 levels and immunomodulators and lymphocytes within LIHC based on the TISIDB database (Fig. 3). Figure 3A illustrates the connection between EZH2 expression and 28 TIICs across cancers. The EZH2 level revealed a positive relationship with type 2 T helper cells (Th2; Spearman: ρ = 0.26, P = 3.17e − 07) and activated CD4 + T cells (Act-cd4; Spearman: ρ = 0.533, P < 2.2e − 16) (Fig. 3B). Immunomodulators are immune inhibitors, immunostimulators, or major histocompatibility complex molecules (MHC). Figure 3C presents the analyses of the correlation between EZH2 expression and 24 types of human tumour immunoinhibitors. EZH2 expression was positively correlated with LAG3 (Spearman: ρ = 0.166, P = 1.32e − 03) and CTLA4 (Spearman: ρ = 0.182, P = 4.1e − 04) (Fig. 3D). Figure 3E reveals the correlation between EZH2 levels and 45 types of immunostimulators. In LIHC, EZH2 expression was positively correlated with MICB (Spearman: ρ = 0.403, P < 2.2e − 16) and CD276 (Spearman: ρ = 0.257, P = 5.19e − 07) (Fig. 3F). As shown in Fig. 3G, EZH2 levels were correlated with 21 MHC molecules across cancers. EZH2 was positively related to TAP1 and HLA-E in LIHC (Fig. 3H). Subsequently, we explored the relationship between EZH2 expression and immune infiltration in 39 tumour types by using the TIMER database (Figure. S1). Cancer survival and LNM were predicted according to TIIC levels [45–47]. EZH2 expression revealed a positive relationship with infiltrating levels of CD8 + T cells in 20 cancer cases, those of macrophages for 12 cancers, those of CD4 + T cells for 18 cancers, those of DCs for 19 cancers, and those of neutrophils for 24 cancers. The results showed that in LIHC, EZH2 expression levels correlated positively with the infiltration degrees of CD4 + T cells (R = 0.378, P = 3.84e-13), CD8 + T cells (R = 0.284, P = 9.30e-08), DCs (R = 0.453, P = 1.38e-18), neutrophils (R = 0.374, P = 7.02e-13), macrophages (R = 0.436, P = 3.22e-17), and B cells (R = 0.474, P = 1.24e-20) (Fig. 4A). The current study suggested that EZH2 stimulated TIIC infiltration within LIHC, especially B cells, neutrophils, CD4 + T cells, DCs, and CD8 + T cells.

### Association of EZH2 expression with immune markers

Subsequently, an association between EZH2 levels with immune infiltration was analysed in 39 cancers derived from the TIMER database. EZH2 expression revealed a positive relationship with the degree of CD8 + T cell infiltration in 20 cancers, macrophages in 12 cancers, CD4 + T cells in 18 cancers, DCs in 19 cancers, and neutrophils in 24 cancers (Additional file 1: Figure S1). Figure 4A showed that EZH2 expression levels correlated positively with the infiltration degrees of CD4 + T cells (R = 0.378, P = 3.84e-13), CD8 + T cells (R = 0.284, P = 9.30e-08), DCs (R = 0.453, P = 1.38e-18), neutrophils (R = 0.374, P = 7.02e-13), macrophages (R = 0.436, P = 3.22e-17), and B cells (R = 0.474, P = 1.24e-20) in LIHC. Moreover, in some cancer types, including THYM, BRCA, HNSC, and KIRC, the immune infiltration degrees were markedly associated with EZH2 (Additional file 1: Figure S1). The results confirmed that EZH2 expression was related to immune markers and the levels of different T cells, TAMs, M1/M2 macrophages, monocytes, and DCs in LIHC. The scatter plot in Fig. 4B–E shows the correlation of EZH2 levels with macrophage phenotype markers (MS4A4A, CD63 for M2 macrophages; IFR5, COX2 for M1 macrophages; IL10, CD68 for TAMs) and monocytes (such as CSF1R and CD86) within LIHC. The TIMER database confirmed that EZH2 expression was markedly related to marker expression in TAMs, M2 macrophages, M1 macrophages, and monocytes (Fig. 4B–E). To investigate the relationship between EZH2 and TIIC levels in LIHC, the TIMER and GEPIA databases were adopted to analyse the connection between EZH2 and immune markers (Tables 2, 3). As shown in Table 2, after purity adjustment, EZH2 expression exhibited a positive relationship with marker expression in T cells and immune cells in HCC. EZH2 upregulation in M2 macrophages was associated with CD8 + T-cell and DC infiltration in LIHC. DCs can cause cancer migration by reducing CD8 + T-cell toxicity and elevating the number of Tregs [48]. In addition, the EZH2 level revealed a positive relationship with markers for Tregs and exhausted T cells (CTLA4, TIM-3, LAG3, Act-CD4, CD276, and PD-1). As shown in Table 3, we verified a similar correlation between EZH2 levels and monocytes, M2 macrophages, and TAM markers in the GEPIA database in LIHC. As a result, immune markers of diverse TAMs, T cells, DCs, monocytes, and M1/M2 macrophages were related to the EZH2 level in LIHC.

**Table 2:** The correlation analysis between EZH2 and related immune cell genes and markers of immune cells in TIMER

Immune cell	Gene markers	None		Purity	
		Cor	*P*-value	Cor	*P*-value
CD8 + T cell	CD8A	0.180	**	0.286	***
	CD8B	0.160	*	0.272	***
T cell	CD6	0.161	*	0.305	***
	CD3D	0.235	***	0.350	***
	CD3E	0.161	*	0.214	***
	SH2D1A	0.147	*	0.265	***
	TRAT1	0.141	*	0.267	***
	CD3G	0.226	***	0.324	***
	CD2	0.182	**	0.323	***
B cell	CD19	0.244	***	0.318	***
	FCRL2	0.173	**	0.303	***
	KIAA0125	0.118	0.023	0.226	***
	TNFRSF17	0.130	0.012	0.264	***
	SPIB	0.373	***	0.464	***
	PNOC	0.166	*	0.279	***
	CD79A	0.131	0.012	0.245	***
Monocyte	CD86	0.294	***	0.449	***
	CD115(CSF1R)	0.153	*	0.296	***
TAM	CD68	0.210	***	0.301	***
	IL10	0.220	***	0.335	***
M1 macrophage	IRF5	0.482	***	0.487	***
	COX2(PTGS2)	0.088	0.092	0.212	***
M2 macrophage	CD163	0.101	0.052	0.218	***
	MS4A4A	0.102	0.051	0.231	***
Neutrophils	FPR1	0.218	***	0.35	***
	SIGLEC5	0.271	***	0.402	***
	CSF3R	0.285	***	0.428	***
	FCGR3B	0.175	**	0.206	**
	CEACAM3	0.16	0.041	0.186	**
	CD116(ITGAM)	0.289	***	0.387	***
Dendritic cell	CCL13	0.137	*	0.196	**
	CD209	0.127	0.014	0.212	***
	HSD11B1	0.332	***	− 0.336	***
	HLA-DPB1	0.154	*	0.271	***
	HLA-DQB1	0.146	*	0.256	***
	HLA-DRA	0.167	*	0.287	***
	HLA-DPA1	0.158	*	0.283	***
	BCDA-1(CD1C)	0.114	0.029	0.207	**
	BDCA-4(NRP1)	0.263	***	0.291	***
	CD11c(ITGAX)	0.348	***	0.484	***
Natural killer cell	XCL2	0.17	**	0.258	***
	KIR2DL1	0.039	0.455	0.018	0.742
	KIR2DL3	0.168	*	0.205	**
	KIR2DL4	0.211	***	0.241	***
Mast cell	TPSB2	− 0.037	0.479	0.001	0.986
	HDC	− 0.149	*	− 0.121	0.025
Th1	IFN-γ(IFNG)	0.249	***	0.333	***
	TNF-α(TNF)	0.282	***	0.414	***
	STAT4	0.238	***	0.303	***
	STAT1	0.387	***	0.246	***
Th2	GATA3	0.172	**	0.309	***
	STAT6	0.193	**	0.179	**
	STAT5A	0.329	***	0.389	***
Tfh	BCL6	0.201	***	0.205	**
	IL21	0.147	*	0.188	**
Th17	STAT3	0.168	*	0.209	***
	IL17A	0.072	0.168	0.075	0.164
Effector T cell	CX3CR1	0.187	**	0.242	***
	FGFBP2	− 0.085	0.103	− 0.07	0.195
	FCGR3A	0.295	***	0.383	***
Effector memory T cell	PD-1 (PDCD1)	0.295	***	0.391	***
	DUSP4	0.256	***	0.363	***
Central memory T cell	CCR7	0.083	0.111	0.219	***
	SELL	0.196	**	0.308	***
	IL7R	0.093	0.073	0.201	**
Resident memory T cell	CD69	0.109	0.035	0.222	***
	ITGAE	0.279	***	0.280	***
	CXCR6	0.156	*	0.288	***
	MYADM	0.354	***	0.388	***
Exhausted T cell	TIM-3 (HAVCR2)	0.310	***	0.472	***
	TIGIT	0.262	***	0.385	***
	LAG3	0.287	***	0.332	***
	CXCL13	0.264	***	0.342	***
	LAYN	0.185	**	0.257	***
Resting treg T cell	FOXP3	0.191	**	0.262	***
	IL2RA	0.252	***	0.377	***
Effector treg T cell	CTLA4	0.296	***	0.404	***
	CCR8	0.383	***	0.479	***

Cor, R-value of Spearman correlation; Purity, tumor purity-adjusted correlation. None, correlation without adjustment. *P < 0.01; **P < 0.001; ***P < 0.0001

**Table 3:** Correlation analysis between EZH2 markers of immune cells in GEPIA2

Immune cell	Gene markers	Tumor		Tumor-sum		Normal		Normal-sum	
		Cor	*P*-value	Cor	*P*-value	Cor	*P*-value	Cor	*P*-value
CD8 + T cell	CD8A	0.2	***	0.18	**	0.71	***	0.69	***
	CD8B	0.15	***			0.62	***		
T cell	CD6	0.029	0.11	0.17	*	0.49	**	0.71	***
	CD3D	0.23	***			0.65	***		
	CD3E	0.13	0.01			0.59	***		
	SH2D1A	0.13	0.011			0.68	***		
	TRAT1	0.034	0.51			0.65	***		
	CD3G	0.24	***			0.63	***		
	CD2	0.15	*			0.52	**		
B cell	CD19	0.11	0.03	0.21	***	0.39	*	0.36	***
	FCRL2	0.076	0.14			0.57	***		
	KIAA0125	0.15	*			0.53	***		
	TNFRSF17	0.094	0.071			0.6	***		
	SPIB	0.14	*			0.43	*		
	PNOC	0.1	0.047			0.59	***		
	CD79A	0.083	0.11			0.51	**		
Monocyte	CD86	0.32	***	0.27	***	0.55	***	0.55	***
	CD115(CSF1R)	0.22	***			0.49	**		
TAM	CD68	0.23	***	0.26	***	0.53	***	0.57	***
	IL10	0.21	***			0.43	*		
M1 Macrophage	IRF5	0.44	***	0.37	***	0.44	*	0.44	*
	COX2(PTGS2)	0.035	0.5			0.27	0.055		
M2 Macrophage	CD163	0.16	*	0.12	0.017	0.38	*	0.47	**
	MS4A4A	0.16	*			0.48	**		
Neutrophils	FPR1	0.24	***	0.35	***	0.31	0.028	0.49	**
	SIGLEC5	0.27	***			0.17	0.24		
	CSF3R	0.21	***			0.5	**		
	FCGR3B	0.03	0.56			0.32	0.021		
	CEACAM3	0.15	*			0.25	0.078		
	CD116(ITGAM)	0.34	***			0.69	***		
Natural killer cell	XCL2	0.049	0.35	0.15	*	0.67	***	0.6	***
	KIR2DL1	0.11	0.038			0.62	***		
	KIR2DL3	0.21	***			0.22	0.12		
	KIR2DL4	0.2	***			0.32	0.025		
Dendritic cell	CCL13	0.065	0.22	0.068	0.19	0.41	*	0.64	***
	CD209	0.16	*			0.44	*		
	HSD11B1	− 0.22	***			0.094	0.51		
	HLA-DPB1	0.22	***			0.58	***		
	HLA-DQB1	0.088	0.09			0.52	***		
	HLA-DRA	0.22	***			0.59	***		
	HLA-DPA1	0.2	**			0.54	***		
	BCDA-1(CD1C)	0.17	**			0.63	***		
	BDCA-4(NRP1)	0.28	***			0.41	*		
	CD11c(ITGAX)	0.25	***			0.5	***		
Mast cell	TPSB2	− 0.042	0.42	0.031	0.56	0.096	0.51	− 0.097	0.5
	HDC	− 0.059	0.26			− 0.083	0.57		
Th1	IFN-γ(IFNG)	0.21	***	0.29	***	0.65	***	0.84	***
	TNF-α(TNF)	0.11	.031			0.33	0.02		
	STAT4	0.081	0.12			0.61	***		
	STAT1	0.21	***			0.8	***		
Th2	GATA3	0.18	**	0.29	***	0.19	0.19	0.62	***
	STAT6	0.18	**			0.57	***		
	STAT5A	0.22	***			0.69	***		
Tfh	BCL6	0.1	0.054	0.17	**	0.011	0.94	0.11	0.46
	IL21	0.12	0.023			0.31	0.031		
Th17	STAT3	0.29	***	0.26	***	0.11	0.44	0.2	0.16
	IL17A	0.0028	0.96			0.25	0.085		
Effector T cell	CX3CR1	0.14	*	0.27	***	0.22	**0.13**	0.45	*
	FGFBP2	− 0.098	0.06			0.074	0.61		
	FCGR3A	0.29	***			0.5	***		
Effector memory T cell	PD-1 (PDCD1)	0.22	***	0.39	***	0.73	***	0.72	***
	DUSP4	0.29	***			0.54	***		
Central memory T cell	CCR7	0.064	0.22	0.12	0.023	0.58	***	0.68	***
	SELL	0.12	0.024			0.54	***		
	IL7R	0.029	0.58			0.56	***		
Resident memory T cell	CD69	0.039	0.46	0.28	***	0.63	***	0.64	***
	ITGAE	0.23	***			0.092	0.52		
	CXCR6	0.14	*			0.44	*		
	MYADM	0.29	***			0.24	0.1		
Exhausted T cell	TIM-3(HAVCR2)	0.13	0.014	0.3	***	0.53	***	0.65	***
	PD-1 (PDCD1)	0.22	***			0.73	***		
	TIGIT	0.22	***			0.61	***		
	LAG3	0.2	**			0.48	**		
	CXCL13	0.097	0.064			0.31	0.028		
	LAYN	0.087	0.095			0.52	***		
Resting treg T cell	FOXP3	− 0.002	0.96	0.15	*	0.42	*	0.6	***
	IL2RA	0.19	**			0.37	*		
Effector treg T cell	CTLA4	0.23	***	0.25	***	0.65	***	0.68	***
	CCR8	0.21	***			0.51	**		
	TNFRSF9	0.015	0.77			0.65	***		

Tumour, correlation analysis in tumour tissue of TCGA; Normal, correlation analysis in normal tissue of TCGA. *P < 0.01; **P < 0.001; ***P < 0.0001

### Expression levels of EZH2 in LIHC

**Fig. 5:**
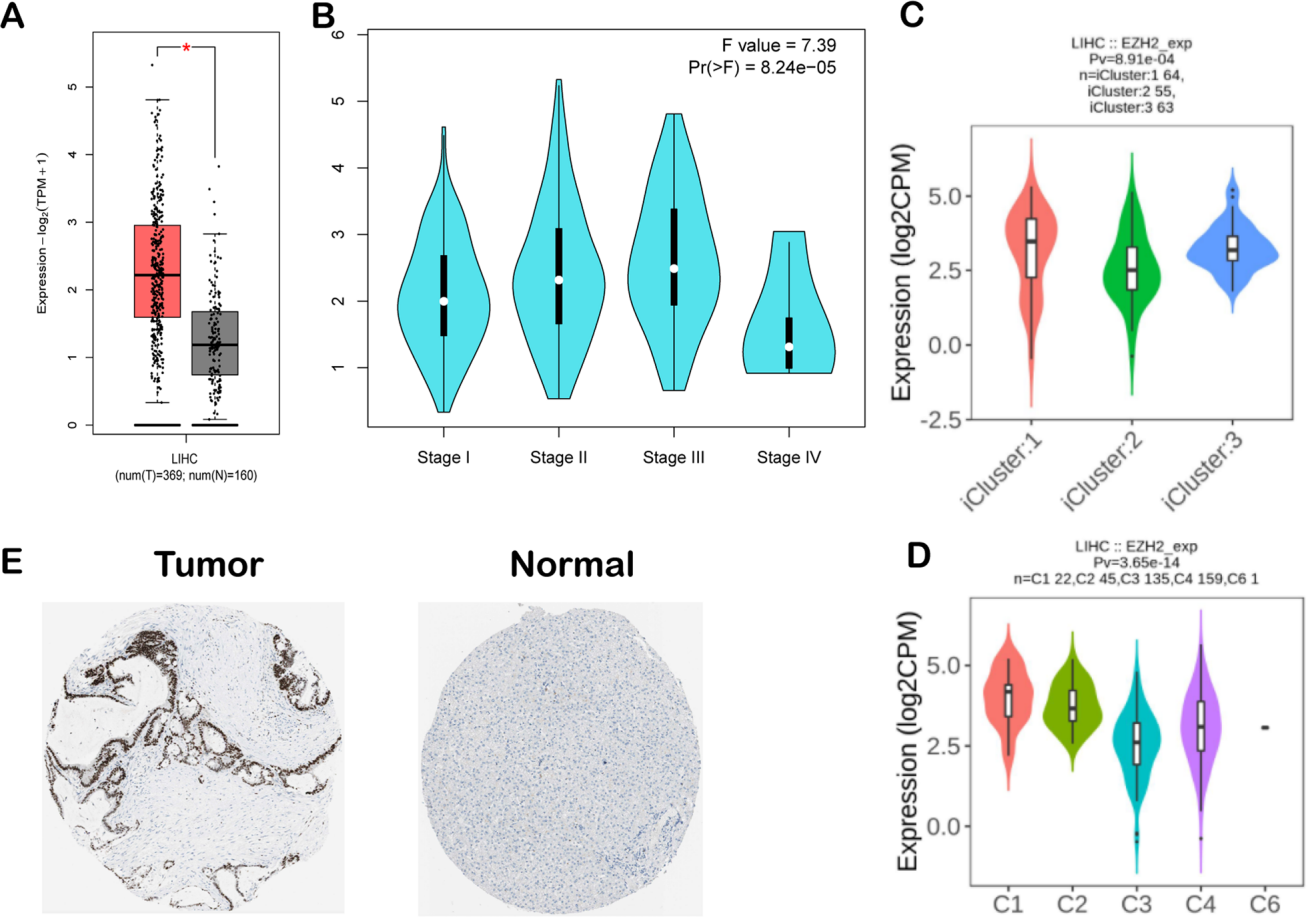
EZH2 expression levels in LIHC. **A** LIHC and healthy samples from the GEPIA database; **B** EZH2 expression in diverse liver hepatocellular carcinoma stages in the GEPIA database; **C** EZH2 levels within diverse LIHC molecular subtypes derived from the TISIDB database; **D** EZH2 expression within diverse LIHC immune subtypes in the TISIDB database; **E** EZH2 protein expression within LIHC samples in relation to healthy samples in the Human Protein Atlas data. T: EZH2 protein expression within cancer samples (quantity: 75–25%; intensity: strong; staining: high) https://www.proteinatlas.org/ENSG00000106462-EZH2/pathology/liver+cancer#img; N: Protein levels of EZH2 in normal tissue (staining: Not detected; intensity: weak, quantity: < 25%) https://www.proteinatlas.org/ENSG00000106462-EZH2/tissue/liver#img;

The GEPIA database demonstrated that the expression level of EZH2 in hepatocellular carcinoma samples increased in relation to healthy controls (Fig. 5A). Furthermore, EZH2 was related to the tumour immune microenvironment (TIME). According to the GEPIA database, compared with different stages of LIHC, the EZH2 expression level was higher in stage III and lower in stage IV (Fig. 5B). Vesteinn Thorsson's study clustered six immune subtypes for cancer and revealed that the immune subtypes of cancer might play a key role in predicting disease outcome [49]. According to our results, we detected that EZH2 expression was the highest in C1 (wound healing) and C2 (IFN-γ), whereas it was the lowest in C3 (inflammation) in TISIDB (Fig. 5D). Moreover, we analysed the correlation between EZH2 expression levels and different molecular subtypes in hepatocellular carcinoma. We discovered EZH2 expression, which was highest in iCluster: 1 type and lowest in iCluster: 2 type, in four molecular types (Fig. 5C). According to the HPA database, the increased staining intensity of the EZH2 protein level was detected in tumour tissues compared with noncarcinoma tissues (Fig. 5E).

### Coexpression genes were correlated with EZH2 in LIHC

**Fig. 6:**
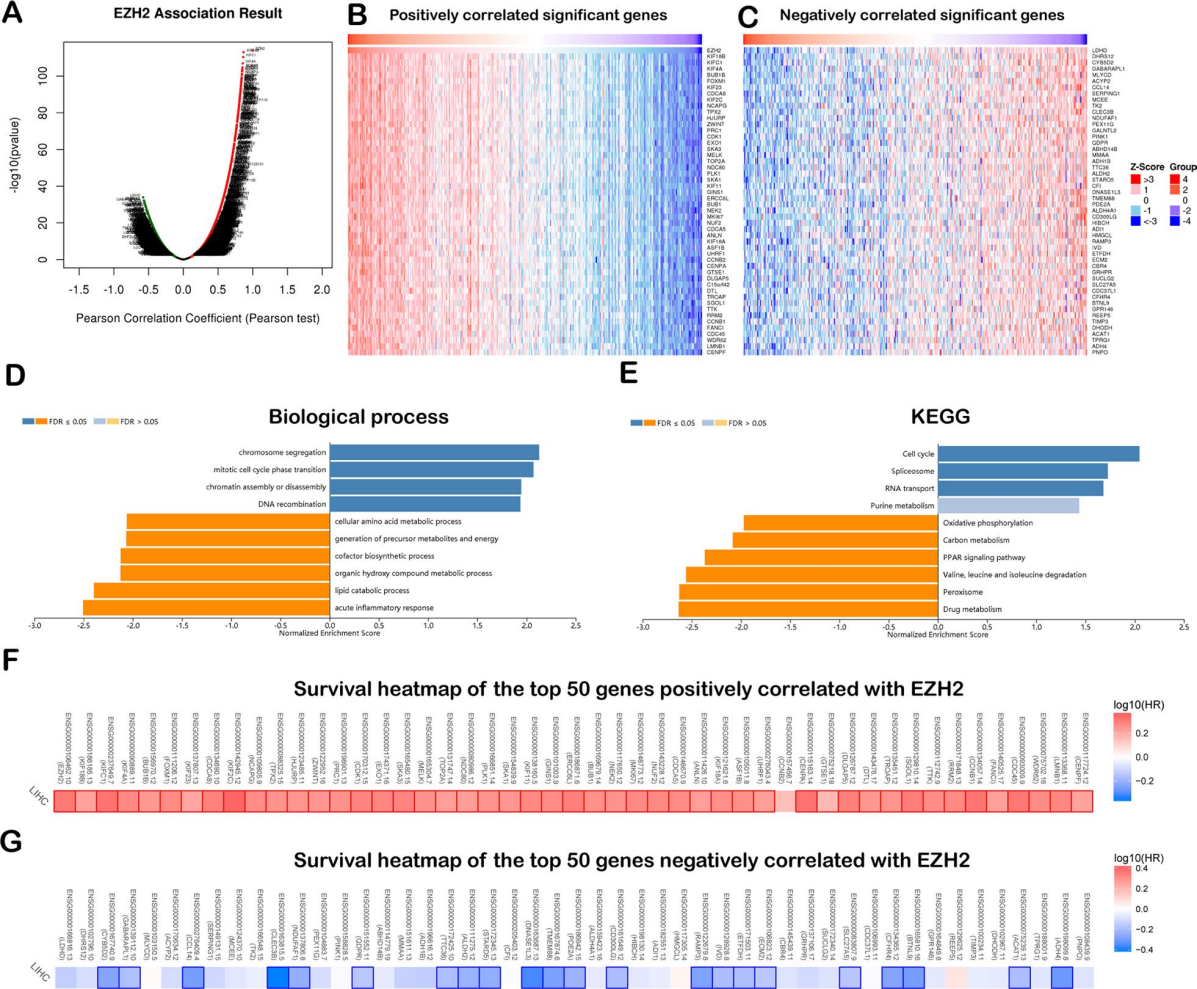
Co-expression genes of EZH2 within LIHC (LinkedOmics). **A** Connection of EZH2 with DEGs in LIHC analysed by the Spearman test. Red and green indicate the positive and negative correlation, respectively; **B** and **C** Heatmap shows the 50 most significantly positive or negative genes within LIHC; **D** and **E** GSEA for analysing the GO annotation and KEGG pathway enriched with EZH2 co-expressed genes in LIHC; **F** Survival heatmaps show the 50 most significantly positive or negative genes within LIHC

The biological effect of EZH2 on LIHC was probably connected with the neighbouring gene expression within LIHC. EZH2 coexpression profiles were checked through the ‘LinkFinder’ module LinkedOmics. The EZH2 coexpression gene levels within 371 LIHC cases were analysed through the LinkedOmics database (Additional file 3: Table S2). We discovered that 12,451 genes were positively related to EZH2, whereas 7,471 were negatively correlated with EZH2 (Fig. 6A). The heatmap presented 50 gene sets positively and negatively correlated with EZH2 (Fig. 6B, C).

### GO and KEGG analysis of EZH2-related coexpressed genes in LIHC

GO analysis conducted using GSEA in LinkedOmics revealed that EZH2 coexpressed genes were mainly related to DNA recombination, mitotic cell cycle phase transition, and chromosome segregation (Fig. 6D). Through KEGG pathway analysis, coexpressed genes revealed a major enrichment of microRNAs in cancer, the cell cycle, the spliceosome, and pyrimidine metabolism (Fig. 6E). The top 50 most remarkably positive genes were the high-risk factors for LIHC, and 49 of them had large HRs (Fig. 6F). By contrast, 22 markedly negative genes had low HRs (Fig. 6G).

## Discussion

EZH2 is a member of PcGs and an epigenetic regulator that can inhibit transcription [16]. EZH2 plays a vital role in cell lineage determination and related signalling pathways and is a master regulator of cell cycle progression, autophagy, apoptosis, DNA damage repair, and cellular senescence inhibition [23–26]. Recent studies have revealed that EHZ2, as a modifier associated with epigenetic regulation and immune function, can promote local and systemic anticancer immunity by modulating TH-1 chemokine levels and affecting MDSC CD8 + T-cell infiltration [27–31]. Hence, our study illustrates that EZH2 expression predicts HCC survival. EZH2 upregulation indicates dismal survival. Consequently, the current study sheds more light on EZH2’s regulatory function in LIHC through comprehensive and systematic analysis and studies.

In the current study, the EZH2 level exhibited a remarkable relationship with lymphocyte infiltration and immune responses in LIHC. Figure 4A shows that EZH2 expression was positively related to the TIIC infiltrating levels of CD4 + T cells, macrophages, CD8 + T cells, B cells, neutrophils, and DCs in LIHC. EZH2 expression was related to gene markers for diverse T cells, M1/M2 macrophages, TAMs, DCs, and monocytes in LIHC (Tables 2, 3). T-cell exhaustion is an important cause of poor antitumor immunity; hence, suppressing such exhaustion is a crucial immunotherapeutic strategy to manage cancer [50–52]. According to the obtained results, EZH2 upregulation revealed a positive correlation with several critical genes associated with exhausted T cells, such as TIM-3, PD-1, and LAG3. These T cells play the role of therapeutic targets for immunotherapy [53, 54]. EZH2 upregulation in M2 macrophages was associated with CD8 + T-cell and DC infiltration in LIHC. DCs can cause cancer migration by decreasing CD8 + T-cell toxicity and elevating the number of Tregs [48]. Our analysis revealed that EZH2 regulates the tumour immune microenvironment in LIHC, which is related to the activation and regulation of B-cell, DC, and T-cell immune responses.

EZH2 mRNA expression levels in normal tissues and liver hepatocellular carcinoma were analysed in detail based on GEPIA, Oncomine, and TISIDB online datasets. The expression level of EZH2 in LIHC was higher than that in normal tissues. Figure 3E displays the correlation between EZH2 levels and 45 types of immunostimulators. The current article assessed the connection between EZH2 and immunity based on the TISIDB database. As a result, EZH2 was closely associated with immunostimulators (MICB, CD276), lymphocytes (activated CD4 T cells, Th2), MHC molecules (including TAP1, HLA-E), and immunoinhibitors (CTLA4, LAG3). However, LIHC can be subdivided into several molecular subtypes. Based on the aforementioned analysis, the TISIDB database revealed that EZH2 exhibited the greatest expression in iCluster: 1, followed by iCluster: 3, and less in iCluster: 2 (Fig. 5D). The EZH2 expression level is a different indistinct immune subtype of hepatocellular carcinoma, and C1 (wound healing) and C2 (IFN-γ) exhibited the highest expression in relation to the remaining four subtypes (Fig. 5C). According to our comprehensive analysis of EZH2 gene expression in LIHC and different databases of different subtypes, EZH2 might be closely related to immunological properties in the microenvironment.

Based on the GEPIA and K-Meier plotter databases, we further analysed EZH2-related survival (OS and RFS) and discovered that EZH2 is a detrimental prognostic factor for LIHC. High EZH2 expression was correlated with a poor prognosis in LIHC. The EZH2 expression level of stage III was higher and that of stage IV, indicating the possible role of EZH2 in liver hepatocellular carcinoma prognosis.

In conclusion, the upregulation of EZH2 was markedly related to TIIC infiltration levels (B cells, CD8 + T cells, CD4 + T cells, DCs, neutrophils, and many functional T cells). EZH2 has an immune-stimulating effect, which may be a critical factor that promotes T-cell exhaustion within LIHC. According to these results, EZH2 has a critical function within the immune microenvironment and deserves to be regarded as a prognostic marker and immunotherapeutic target for hepatocellular carcinoma.

## Supplementary Information

**Additional file1: Figure S1**. Correlation of EZH2 expression with immune infiltration level in different tumor types via TIMER database.

**Additional file2: Table S1**. EZH2 expression in cancers verus normal tissue in oncomine database.

**Additional file3: Table S2**. The co-expressed genes of EZH2 in LIHC.

## Data Availability

The data that support the findings of this study are available in TIMER (https://cistrome.shinyapps.io/timer/), OnCoLnc Database (http://www.oncolnc.org/), Kaplan–Meier Plotter (http://kmplot.com/), GEPIA2 (http://gepia2.cancer-pku.cn/), KEGG database (https://www.kegg.jp/), The HPA database (https://www.proteinatlas.org/) and LinkedOmics Database (http://www.linkedomics.org).

## Abbreviations

EZH2: Enhancer of zeste homolog 2
LIHC: Liver hepatocellular carcinoma
KM: Kaplan–Meier
DC: Dendritic cell
SCNA: Somatic copy number alteration
FDR: False discovery rate
FC: Fold-change
LNM: Lymph node metastasis
TME: Tumor microenvironment
