# Supplementary material for: The correlation of EZH2 expression with the progression and prognosis of hepatocellular carcinoma
Source: BMC Immunol. 2022 Jun 4;23:28. doi: 10.1186/s12865-022-00502-7 (PMC9166340; doi:10.1186/s12865-022-00502-7)
Supplement: Supplementary file 1 — Additional file1: Figure S1. Correlation of EZH2 expression with immune infiltration level in different tumor types via TIMER database. [file 12865_2022_502_MOESM1_ESM.pdf]

Supplementary Figure 1 Correlation of EZH2 expression with immune infiltration level in different tumor types via TIMER database.

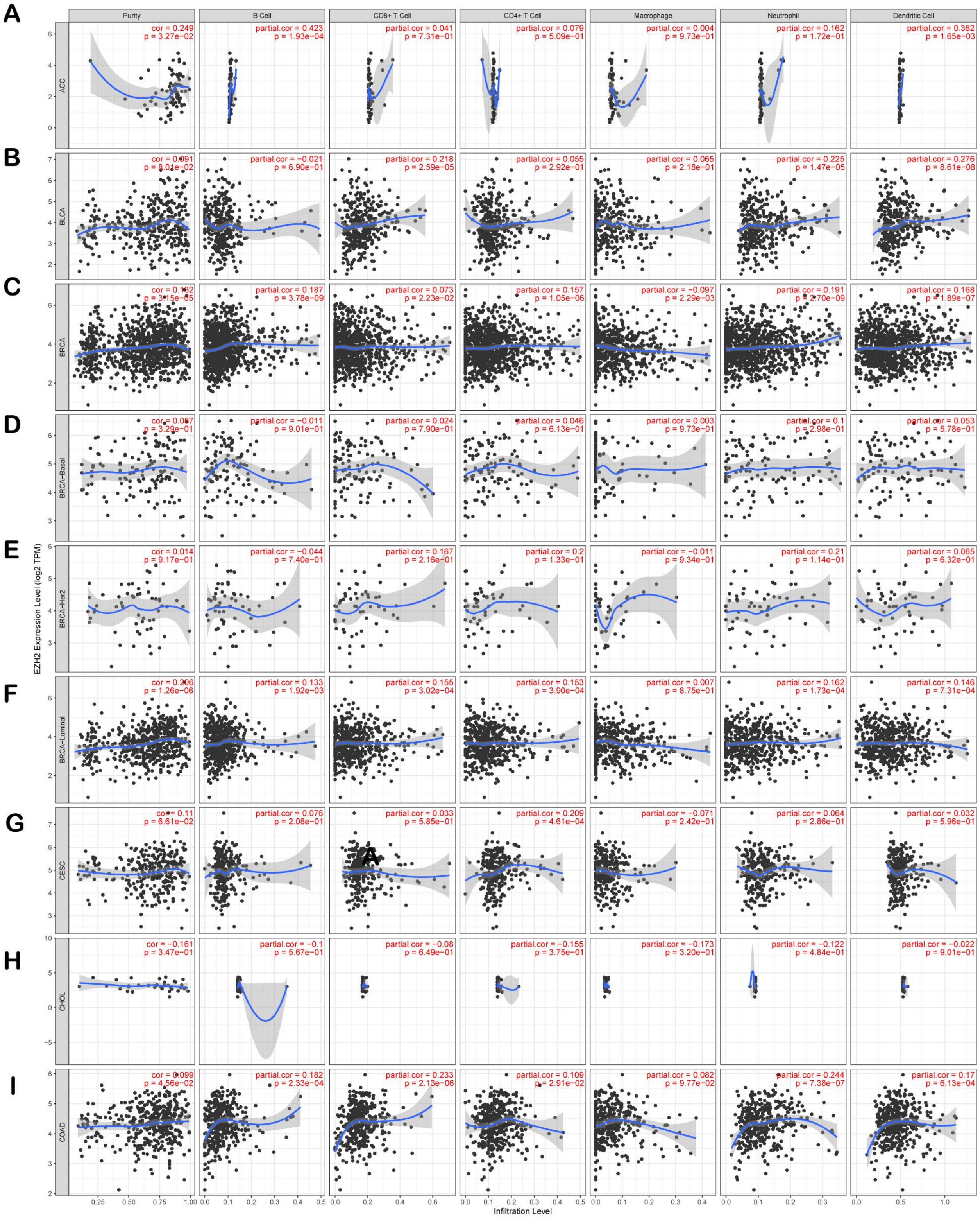

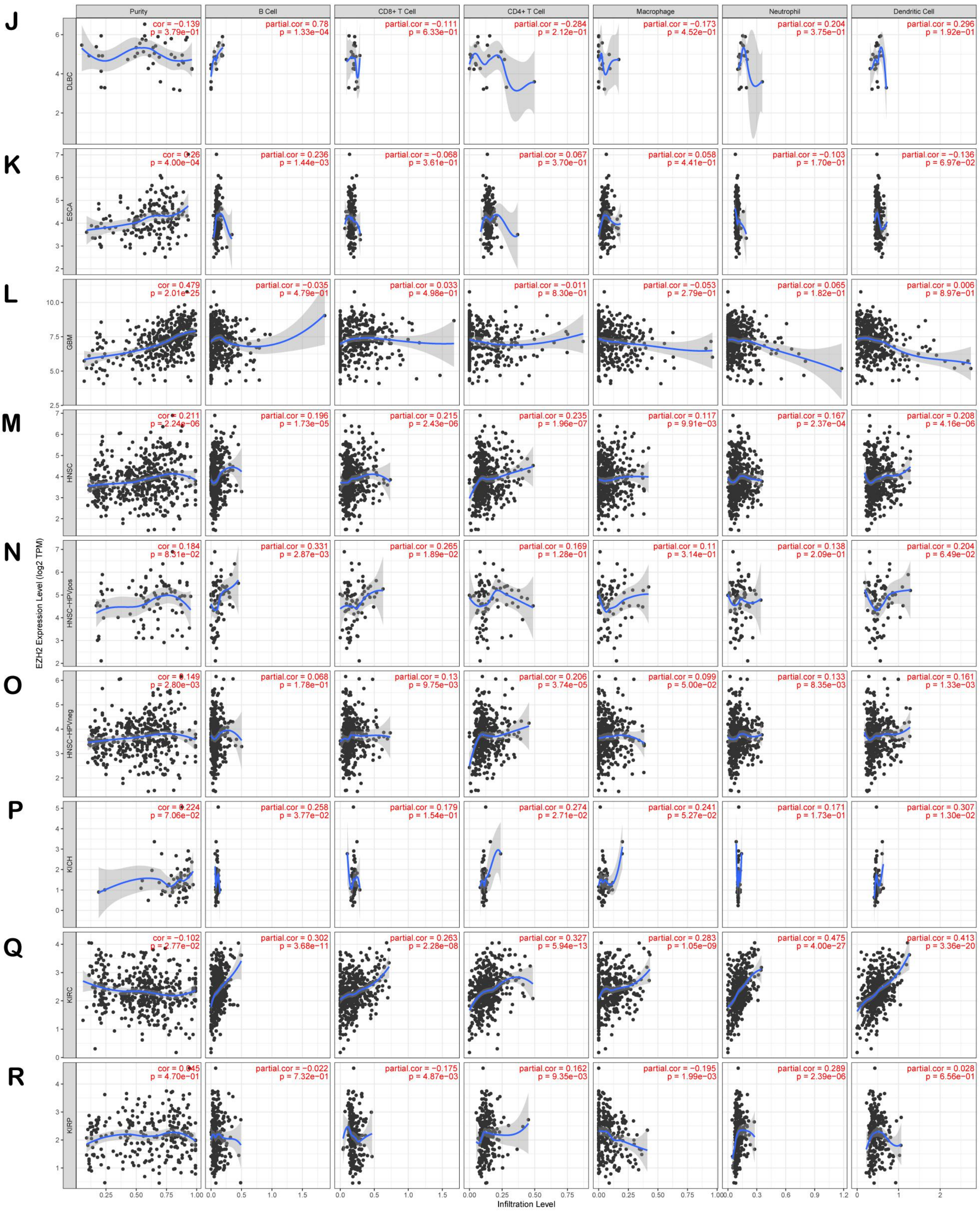

J
K
L
M
N
O
P
Q
R
Purity
B Cell
CD8+ T Cell
CD4+ T Cell
Macrophage
Neutrophil
Dendritic Cell
DLBC
ESCA
GBM
HNSC
HNSC−HPVpos
HNSC−HPVneg
KICH
KIRC
KIRP
EZH2 Expression Level (log2 TPM)
Infiltration Level
cor = −0.139 p = 3.79e−01
partial.cor = 0.78 p = 1.33e−04
partial.cor = −0.111 p = 6.33e−01
partial.cor = −0.284 p = 2.12e−01
partial.cor = −0.173 p = 4.52e−01
partial.cor = 0.204 p = 3.75e−01
partial.cor = 0.296 p = 1.92e−01
p = 4.00e−04
partial.cor = 0.236 p = 1.44e−03
partial.cor = −0.068 p = 3.61e−01
partial.cor = 0.067 p = 3.70e−01
partial.cor = 0.058 p = 4.41e−01
partial.cor = −0.103 p = 1.70e−01
partial.cor = −0.136 p = 6.97e−02
cor = 0.479
partial.cor = −0.035 p = 4.79e−01
partial.cor = 0.033 p = 4.98e−01
partial.cor = −0.011 p = 8.30e−01
partial.cor = −0.053 p = 2.79e−01
partial.cor = 0.065 p = 1.82e−01
partial.cor = 0.006 p = 8.97e−01
partial.cor = 0.196 p = 1.73e−05
partial.cor = 0.215 p = 2.43e−06
partial.cor = 0.235 p = 1.96e−07
partial.cor = 0.117 p = 9.91e−03
partial.cor = 0.167 p = 2.37e−04
partial.cor = 0.208 p = 4.16e−06
cor = 0.184
partial.cor = 0.331 p = 2.87e−03
partial.cor = 0.265 p = 1.89e−02
partial.cor = 0.169 p = 1.28e−01
partial.cor = 0.11 p = 3.14e−01
partial.cor = 0.138 p = 2.09e−01
partial.cor = 0.204 p = 6.49e−02
partial.cor = 0.068 p = 1.78e−01
partial.cor = 0.13 p = 9.75e−03
partial.cor = 0.206 p = 3.74e−05
partial.cor = 0.099 p = 5.00e−02
partial.cor = 0.133 p = 8.35e−03
partial.cor = 0.161 p = 1.33e−03
partial.cor = 0.258 p = 3.77e−02
partial.cor = 0.179 p = 1.54e−01
partial.cor = 0.274 p = 2.71e−02
partial.cor = 0.241 p = 5.27e−02
partial.cor = 0.171 p = 1.73e−01
partial.cor = 0.307 p = 1.30e−02
cor = −0.102
partial.cor = 0.302 p = 3.68e−11
partial.cor = 0.263 p = 2.28e−08
partial.cor = 0.327 p = 5.94e−13
partial.cor = 0.283 p = 1.05e−09
partial.cor = 0.475 p = 4.00e−27
partial.cor = 0.413 p = 3.36e−20
p = 4.70e−01
partial.cor = −0.022 p = 7.32e−01
partial.cor = −0.175 p = 4.87e−03
partial.cor = 0.162 p = 9.35e−03
partial.cor = −0.195 p = 1.99e−03
partial.cor = 0.289 p = 2.39e−06
partial.cor = 0.028 p = 6.56e−01

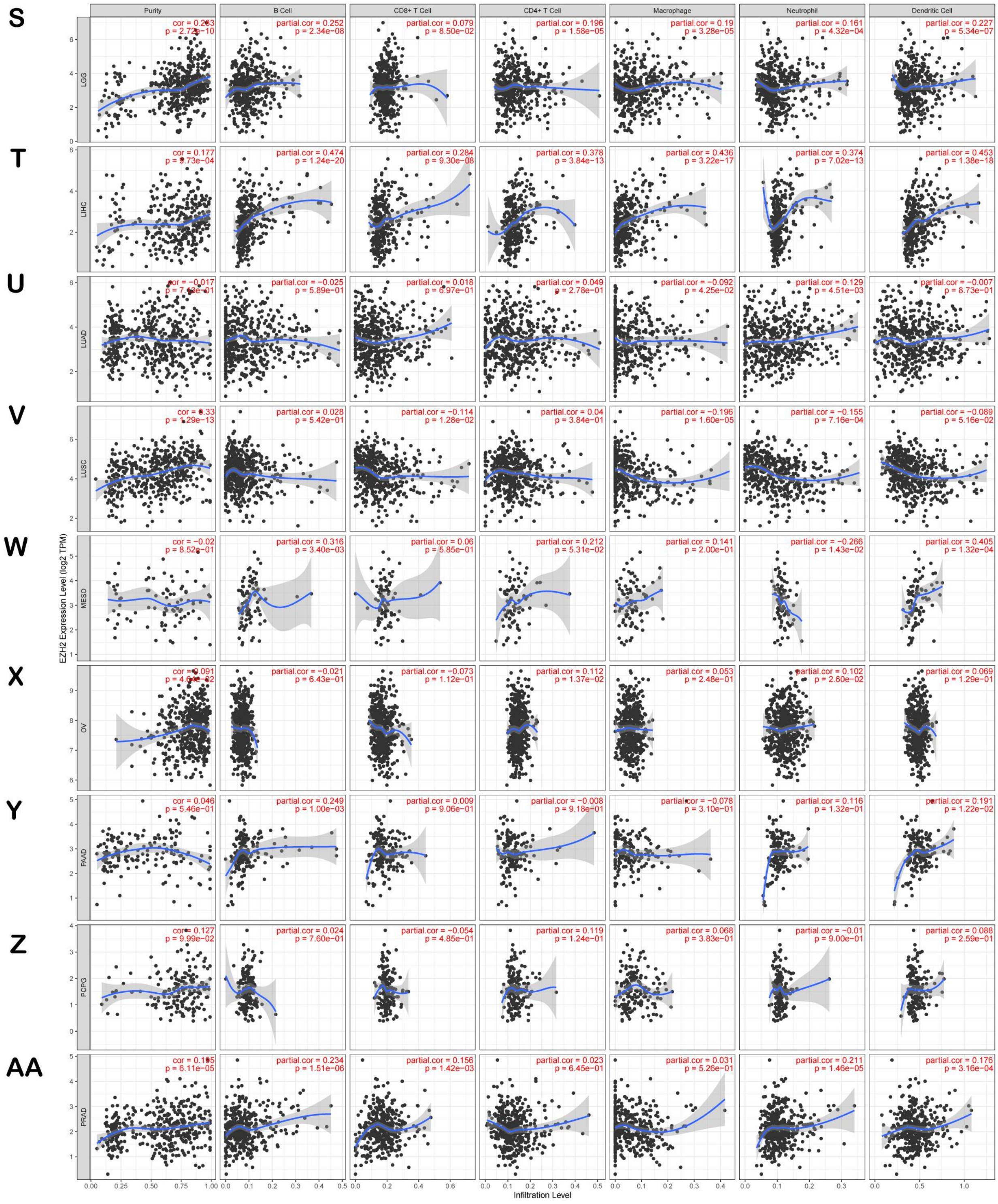
S
T
U
V
W
X
Y
Z
AA
Purity
B Cell
CD8+ T Cell
CD4+ T Cell
Macrophage
Neutrophil
Dendritic Cell
LGG
LIHC
LUAD
LUSC
MESO
OV
PAAD
PCPG
PRAD
EZH2 Expression Level (log2 TPM)
Infiltration Level
partial.cor = 0.252 p = 2.34e−08
partial.cor = 0.079 p = 8.50e−02
partial.cor = 0.196 p = 1.58e−05
partial.cor = 0.19 p = 3.28e−05
partial.cor = 0.161 p = 4.32e−04
partial.cor = 0.227 p = 5.34e−07
cor = 0.177 p = 3.73e−04
partial.cor = 0.474 p = 1.24e−20
partial.cor = 0.284 p = 9.30e−08
partial.cor = 0.378 p = 3.84e−13
partial.cor = 0.436 p = 3.22e−17
partial.cor = 0.374 p = 7.02e−13
partial.cor = 0.453 p = 1.38e−18
partial.cor = −0.025 p = 5.89e−01
partial.cor = 0.049 p = 2.78e−01
partial.cor = −0.092 p = 4.25e−02
partial.cor = 0.129 p = 4.51e−03
partial.cor = −0.007 p = 8.73e−01
partial.cor = 0.028 p = 5.42e−01
partial.cor = −0.114 p = 1.28e−02
partial.cor = 0.04 p = 3.84e−01
partial.cor = −0.196 p = 1.60e−05
partial.cor = −0.155 p = 7.16e−04
partial.cor = −0.089 p = 5.16e−02
partial.cor = 0.316 p = 3.40e−03
partial.cor = 0.06 p = 5.85e−01
partial.cor = 0.212 p = 5.31e−02
partial.cor = 0.141 p = 2.00e−01
partial.cor = −0.266 p = 1.43e−02
partial.cor = 0.405 p = 1.32e−04
partial.cor = −0.021 p = 6.43e−01
partial.cor = −0.073 p = 1.12e−01
partial.cor = 0.112 p = 1.37e−02
partial.cor = 0.053 p = 2.48e−01
partial.cor = 0.069 p = 1.29e−01
cor = 0.046 p = 5.46e−01
partial.cor = 0.249 p = 1.00e−03
partial.cor = 0.009 p = 9.06e−01
partial.cor = −0.008 p = 9.18e−01
partial.cor = 0.116 p = 1.32e−01
partial.cor = 0.024 p = 7.60e−01
partial.cor = −0.054 p = 4.85e−01
partial.cor = 0.119 p = 1.24e−01
partial.cor = 0.068 p = 3.83e−01
partial.cor = −0.01 p = 9.00e−01
partial.cor = 0.088 p = 2.59e−01
p = 6.11e−05
partial.cor = 0.234 p = 1.51e−06
partial.cor = 0.156 p = 1.42e−03
partial.cor = 0.023 p = 6.45e−01
partial.cor = 0.031 p = 5.26e−01
partial.cor = 0.211 p = 1.46e−05
partial.cor = 0.176 p = 3.16e−04

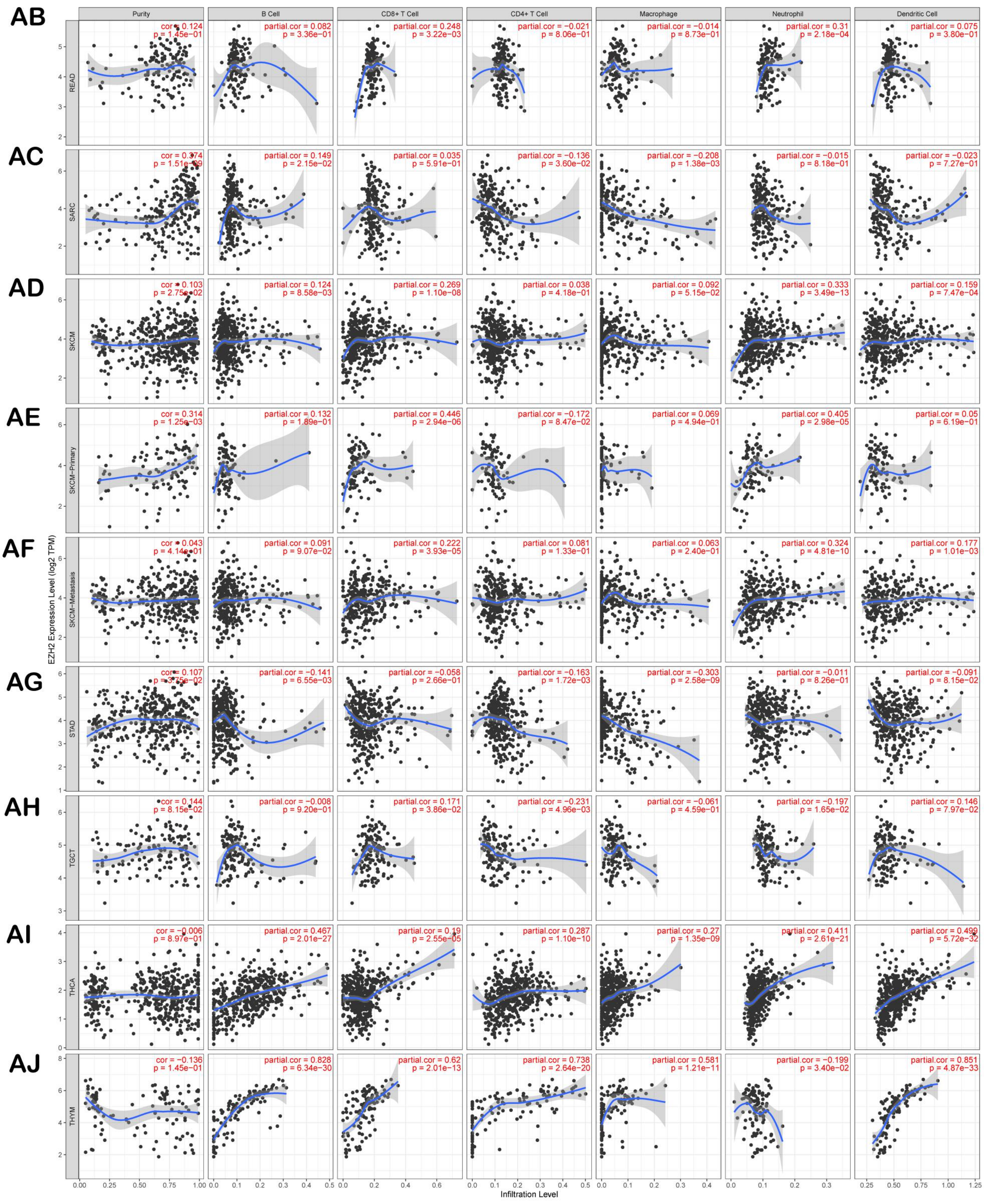

Purity
B Cell
CD8+ T Cell
CD4+ T Cell
Macrophage
Neutrophil
Dendritic Cell
AB
READ
cor = 0.124 p = 1.45e-01
partial.cor = 0.082 p = 3.36e-01
partial.cor = 0.248 p = 3.22e-03
partial.cor = −0.021 p = 8.06e-01
partial.cor = −0.014 p = 8.73e-01
partial.cor = 0.31 p = 2.18e-04
partial.cor = 0.075 p = 3.80e-01
AC
SARC
cor = 0.374 p = 1.51e-09
partial.cor = 0.149 p = 2.15e-02
partial.cor = 0.035 p = 5.91e-01
partial.cor = −0.136 p = 3.60e-02
partial.cor = −0.208 p = 1.38e-03
partial.cor = −0.015 p = 8.18e-01
partial.cor = −0.023 p = 7.27e-01
AD
SKCM
cor = 0.103 p = 2.75e-02
partial.cor = 0.124 p = 8.58e-03
partial.cor = 0.269 p = 1.10e-08
partial.cor = 0.038 p = 4.18e-01
partial.cor = 0.092 p = 5.15e-02
partial.cor = 0.333 p = 3.49e-13
partial.cor = 0.159 p = 7.47e-04
AE
SKCM−Primary
cor = 0.314 p = 1.25e-03
partial.cor = 0.132 p = 1.89e-01
partial.cor = 0.446 p = 2.94e-06
partial.cor = −0.172 p = 8.47e-02
partial.cor = 0.069 p = 4.94e-01
partial.cor = 0.405 p = 2.98e-05
partial.cor = 0.05 p = 6.19e-01
AF
SKCM−Metastasis
cor = 0.043 p = 4.14e-01
partial.cor = 0.091 p = 9.07e-02
partial.cor = 0.222 p = 3.93e-05
partial.cor = 0.081 p = 1.33e-01
partial.cor = 0.063 p = 2.40e-01
partial.cor = 0.324 p = 4.81e-10
partial.cor = 0.177 p = 1.01e-03
AG
STAD
cor = 0.107 p = 3.75e-02
partial.cor = −0.141 p = 6.55e-03
partial.cor = −0.058 p = 2.66e-01
partial.cor = −0.163 p = 1.72e-03
partial.cor = −0.303 p = 2.58e-09
partial.cor = −0.011 p = 8.26e-01
partial.cor = −0.091 p = 8.15e-02
AH
TGCT
cor = 0.144 p = 8.15e-02
partial.cor = −0.008 p = 9.20e-01
partial.cor = 0.171 p = 3.86e-02
partial.cor = −0.231 p = 4.96e-03
partial.cor = −0.061 p = 4.59e-01
partial.cor = −0.197 p = 1.65e-02
partial.cor = 0.146 p = 7.97e-02
AI
THCA
cor = −0.006 p = 8.97e-01
partial.cor = 0.467 p = 2.01e-27
partial.cor = 0.19 p = 2.55e-05
partial.cor = 0.287 p = 1.10e-10
partial.cor = 0.27 p = 1.35e-09
partial.cor = 0.411 p = 2.61e-21
partial.cor = 0.499 p = 5.72e-32
AJ
THYM
cor = −0.136 p = 1.45e-01
partial.cor = 0.828 p = 6.34e-30
partial.cor = 0.62 p = 2.01e-13
partial.cor = 0.738 p = 2.64e-20
partial.cor = 0.581 p = 1.21e-11
partial.cor = −0.199 p = 3.40e-02
partial.cor = 0.851 p = 4.87e-33
EZH2 Expression Level (log2 TPM)
Infiltration Level

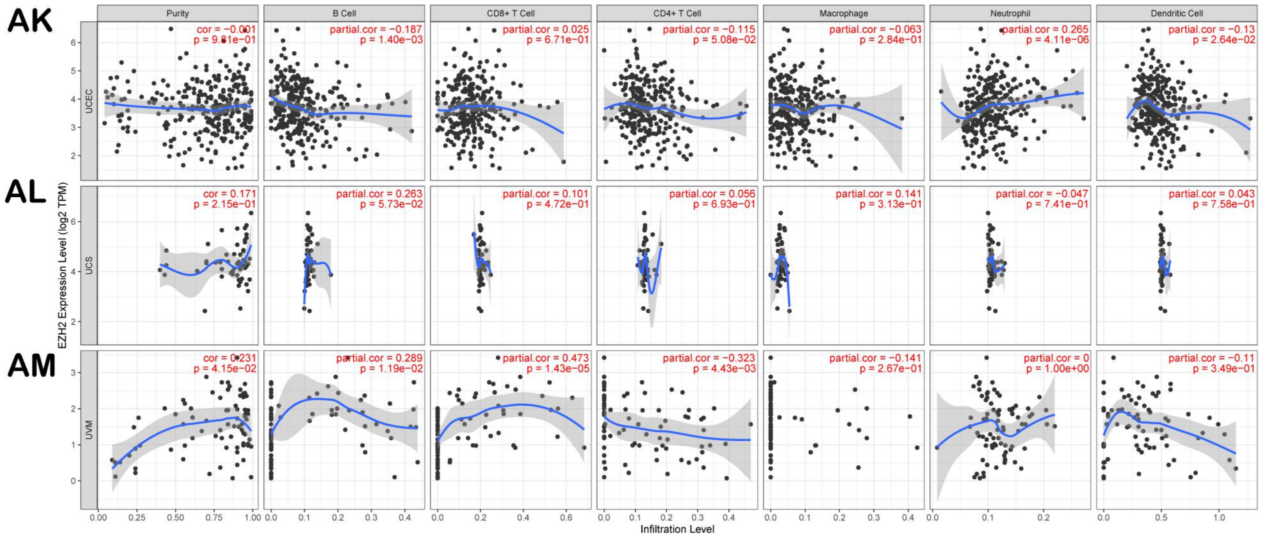

AK
AL
AM
Purity
B Cell
CD8+ T Cell
CD4+ T Cell
Macrophage
Neutrophil
Dendritic Cell
UCEC
UCS
UVM
EZH2 Expression Level (log2 TPM)
Infiltration Level
cor = −0.001
p = 9.81e−01
partial.cor = −0.187
p = 1.40e−03
partial.cor = 0.025
p = 6.71e−01
partial.cor = −0.115
p = 5.08e−02
partial.cor = −0.063
p = 2.84e−01
partial.cor = 0.265
p = 4.11e−06
partial.cor = −0.13
p = 2.64e−02
cor = 0.171
p = 2.15e−01
partial.cor = 0.263
p = 5.73e−02
partial.cor = 0.101
p = 4.72e−01
partial.cor = 0.056
p = 6.93e−01
partial.cor = 0.141
p = 3.13e−01
partial.cor = −0.047
p = 7.41e−01
partial.cor = 0.043
p = 7.58e−01
cor = 0.231
p = 4.15e−02
partial.cor = 0.289
p = 1.19e−02
partial.cor = 0.473
p = 1.43e−05
partial.cor = −0.323
p = 4.43e−03
partial.cor = −0.141
p = 2.67e−01
partial.cor = 0
p = 1.00e+00
partial.cor = −0.11
p = 3.49e−01
