# Supplementary material for: The correlation of EZH2 expression with the progression and prognosis of hepatocellular carcinoma
Source: BMC Immunol. 2022 Jun 4;23:28. doi: 10.1186/s12865-022-00502-7 (PMC9166340; doi:10.1186/s12865-022-00502-7)
Supplement: Supplementary file 2 — Additional file2: Table S1. EZH2 expression in cancers verus normal tissue in oncomine database. [file 12865_2022_502_MOESM2_ESM.pdf]

Supplementary Table 1. EZH2 expression in cancers verus normal tissue in oncomine database.

Cancer	Cancer type	P-value	Fold change	Rank (%)	Sample	Reference (PMID)
Bladder cancer	Infiltrating Bladder Urothelial Carcinoma	1.55E-16	5.294	1%	157	16432078
	Infiltrating Bladder Urothelial Carcinoma	4.14E-5	2.110	9%	60	15173019
Brain and CNS cancer	Brain Glioblastoma	2.80E-38	9.853	1%	557	TCGA
	Anaplastic Astrocytoma	9.74E-14	5.854	1%	180	16616334
	Oligodendroglioma	2.59E-13	4.474	1%	180	16616334
	Glioblastoma	3.03E-18	7.432	2%	180	16616334
	Anaplastic Oligodendroglioma	3.08E-11	5.491	1%	33	16357140
	Glioblastoma	1.64E-8	10.372	3%	84	18565887
Breast cancer	Medullary Breast Carcinoma	7.14E-16	2.848	1%	2136	22522925
	Invasive Breast Carcinoma	2.54E-29	3.797	1%	593	TCGA
	Invasive Lobular Breast Carcinoma	9.65E-17	3.173	1%	593	TCGA
	Male Breast Carcinoma	3.11E-11	2.085	1%	593	TCGA
	Invasive Ductal Breast Carcinoma	1.90E-40	4.501	1%	593	TCGA
	Mixed Lobular and Ductal Breast Carcinoma	1.17E-5	2.241	2%	593	TCGA
	Invasive Ductal and Lobular Carcinoma	7.46E-5	4.140	4%	593	TCGA
	Ductal Breast Carcinoma	2.30E-8	8.874	2%	47	16473279
Cervical cancer	Cervical Squamous Cell Carcinoma Epithelia	3.42E-9	2.649	1%	41	17974957
	Cervical Squamous Cell Carcinoma	6.27E-11	3.552	1%	66	18506748
	Cervical Cancer	1.02E-10	2.837	1%	123	17640062
Colorectal cancer	Colon Adenoma	1.46E-22	3.631	4%	64	18171984
	Cecum Adenocarcinoma	2.17E-13	2.249	2%	237	TCGA
	Rectal Mucinous Adenocarcinoma	2.22E-6	2.283	2%	237	TCGA
	Colon Mucinous Adenocarcinoma	4.74E-11	2.089	3%	237	TCGA
	Rectal Adenocarcinoma	3.69E-16	2.247	5%	237	TCGA
	Colon Adenocarcinoma	4.09E-16	2.349	5%	237	TCGA
	Rectal Adenoma	1.76E-6	2.607	4%	64	18171984
	Colon Adenoma	5.36E-10	2.154	8%	64	18171984
	Colon Adenoma	1.98E-6	2.821	4%	40	20957034
	Colon Carcinoma	6.78E-7	2.742	8%	40	20957034
	Colorectal Carcinoma	1.28E-9	2.163	6%	82	20143136
	Colorectal Carcinoma	1.04E-6	2.201	8%	105	20957034
Esophageal cancer	Esophageal Squamous Cell Carcinoma	3.77E-6	2.098	5%	34	20955586
Gastric cancer	Gastric Intestinal Type Adenocarcinoma	1.89E-10	2.506	3%	69	19081245
Head and neck cancer	Nasopharyngeal Carcinoma	1.38E-5	2.802	4%	41	16912175
Kidney Cancer	Renal Pelvis Urothelial Carcinoma	1.02E-17	6.766	1%	92	16115910
	Hereditary Clear Cell Renal Cell Carcinoma	1.85E-14	2.527	1%	70	19470766
	Non-Hereditary Clear Cell Renal Cell Carcinoma	9.48E-10	2.557	2%	70	19470766

Liver cancer	Hepatocellular Carcinoma	1.52E-68	3.420	2%	445	21159642
	Hepatocellular Carcinoma	6.33E-9	3.296	2%	43	21159642
	Hepatocellular Carcinoma	9.41E-7	5.621	3%	75	17393520
Lung cancer	Small Cell Lung Carcinoma	6.01E-9	20.627	1%	203	11707567
	Squamous Cell Lung Carcinoma	6.43E-8	16.342	1%	203	11707567
	Lung Adenocarcinoma	1.29E-8	3.578	1%	66	17540040
	Squamous Cell Lung Carcinoma	4.45E-16	6.474	2%	156	20421987
	Lung Adenocarcinoma	2.29E-13	3.189	2%	156	20421987
	Large Cell Lung Carcinoma	1.65E-6	6.418	5%	156	20421987
	Lung Adenocarcinoma	6.52E-7	2.554	3%	39	16314486
	Lung Adenocarcinoma	3.15E-12	2.017	5%	107	18297132
	Lung Adenocarcinoma	9.19E-13	2.223	4%	246	22080568
Other cancer	Vulvar Intraepithelial Neoplasia	1.74E-9	4.626	1%	19	17471573
	Adrenal Cortex Carcinoma	1.76E-5	4.733	1%	19	12547710
	Pleural Malignant Mesothelioma	1.03E-5	3.522	3%	54	15920167
	Malignant Fibrous Histiocytoma	1.60E-5	6.966	3%	54	15994966
	Skin Squamous Cell Carcinoma	8.97E-5	2.112	4%	87	18442402
Ovarian cancer	Ovarian Serous Adenocarcinoma	4.15E-7	5.867	4%	53	1948012
	Ovarian Serous Cystadenocarcinoma	1.44E-6	6.584	5%	594	TCGA
Pancreatic cancer	Pancreatic Carcinoma	4.78E-7	2.313	3%	52	19732725
Prostate cancer	Prostate Carcinoma	3.94E-5	2.241	1%	19	16286247
Sarcoma	Pleomorphic Liposarcoma	1.11E-7	10.983	1%	54	15994966
	Round Cell Liposarcoma	2.52E-7	14.690	1%	54	15994966
	Leiomyosarcoma	3.30E-7	14.336	1%	54	15994966
	Synovial Sarcoma	8.38E-7	7.922	1%	54	15994966
	Fibrosarcoma	1.93E-6	9.074	1%	54	15994966
	Malignant Fibrous Histiocytoma	1.60E-5	6.966	3%	54	15994966
	Dedifferentiated Liposarcoma	4.24E-17	2.475	1%	158	20601955
	Pleomorphic Liposarcoma	2.38E-11	4.489	1%	158	20601955
	Myxoid/Round Cell Liposarcoma	2.88E-11	4.892	1%	158	20601955
	Myxofibrosarcoma	1.42E-13	3.756	1%	158	20601955
	Leiomyosarcoma	2.04E-11	4.979	2%	158	20601955
